# Pipoxolan Ameliorates Cerebral Ischemia via Inhibition of Neuronal Apoptosis and Intimal Hyperplasia through Attenuation of VSMC Migration and Modulation of Matrix Metalloproteinase-2/9 and Ras/MEK/ERK Signaling Pathways

**DOI:** 10.1371/journal.pone.0075654

**Published:** 2013-09-24

**Authors:** Yuh-Fung Chen, Huei-Yann Tsai, Kuo-Jen Wu, Lian-Ru Siao, W. Gibson Wood

**Affiliations:** 1 Department of Pharmacology, College of Medicine, China Medical University, Taichung, Taiwan; 2 Department of Pharmacy, China Medical University Hospital, Taichung, Taiwan; 3 Department of Pharmacology, University of Minnesota and Geriatric Research, Education and Clinical Center, VA Medical Center, Minneapolis, Minnesota, United States of America; National Center for Scientific Research Demokritos, Greece

## Abstract

Pipoxolan (PIPO) has anti-spasmodic effects, and it is used clinically to relieve smooth muscle spasms. Cerebrovascular disease is one of the leading causes of disability and death worldwide. The main aim of this study was to investigate the effects of PIPO on cerebral ischemia and vascular smooth muscle cell (VSMC) migration *in vivo* and *in vitro*. Cerebral infarction area, ratio of intima to media area (I/M ratio) and PCNA antibody staining of the carotid artery *in vivo* were measured. Cell viability of A7r5 cells, PDGF-BB-stimulated cell migration, and potential mechanisms of PIPO were evaluated by wound healing, transwell and Western blotting. PIPO (10 and 30 mg/kg p.o.) reduced: the cerebral infarction area; neurological deficit; TUNEL-positive cells; cleaved caspase 3-positive cells; intimal hyperplasia; and inhibited proliferating cell nuclear antigen (PCNA)-positive cells in rodents. PIPO (5, 10 and 15 µM) significantly inhibited PDGF-BB-stimulated VSMC migration and reduced Ras, MEK, and p-ERK levels. Moreover, PIPO decreased levels of matrix metalloproteinases -2 and -9 in PDGF-BB-stimulated A7r5 cells. In summary, PIPO is protective in models of ischemia/reperfusion-induced cerebral infarction, carotid artery ligation-induced intimal hyperplasia and VSMC migration both *in vivo* and *in vitro*. PIPO could be potentially efficacious in preventing cerebrovascular and vascular diseases.

## Introduction

Cerebrovascular disease is one of the leading causes of disability and death. Atherosclerotic stenosis or occlusion of a major cerebral artery is a leading cause of stroke [1-3]. Several experimental models have been developed for the study of cerebral ischemia. A widely used method of inducing cerebral ischemia is to ligate surgically one or both common carotid arteries [4-6].

Blood vessel injury or atherosclerosis usually results in intimal thickening. The migration and proliferation of vascular smooth muscle cells (VSMCs) impact the final size of intimal thickening [7]. Migration of VSMCs plays a crucial role both in atherosclerosis and restenosis after balloon injury. In damaged vessels, VSMCs migrate from the membrane into the intima and induce intimal hyperplasia [8,9]. Abnormal VSMC proliferation and migration cause thickening of endothelial cells result in restenosis after balloon angioplasty or coronary stenting [9-11]. Platelet-derived growth factor (PDGF) from VSMC can cause cell migration, which results in intimal hyperplasia and restenosis formation [12,13]. VSMC migration requires the breakdown of the extracellular matrix. A possible mechanism of VSMC migration is via the secretion of matrix metalloproteinases (MMPs) [14,15]. MMPs are increased at the site of vascular injury [16], and inhibitors of MMPs reduce VSMC migration after vascular injury both *in vitro* and *in vivo* [17]. To date, vascular injury can only be controlled by medication or surgery, but restenosis can occur months or years after injury and initial treatment. A drug that inhibits VSMC migration could be beneficial in treating vascular diseases.

Pipoxolan HCl (5,5-diphenyl-2-[2’-piperidino-ethyl]-3-dioxolane-4-one hydro- chloride), a 1,3-dioxolane derivative also known as rowapraxin, was originally synthesized by Pailer et al. in 1968 [18]. Pipoxolan (PIPO) was effective in treating dysmenorrhea, renal colic, bilateral urinary lithiasis, cholelithiasis, chronic gastritis, post-natal uterine pain, urolithiasis and hydronephrosis [19-21]. PIPO has also been used as an anti-spasmodic medication for relief of smooth muscle spasms in the digestive tract, bronchial tree, urinary tract, and gynecological system [22,23]. PIPO was found to inhibit norepinephrine- and high K^+^- induced vasoconstriction in the rat isolated aortic artery (our unpublished data). The purpose of this study was to determine if PIPO would have protective effects in models of ischemia/reperfusion-induced cerebral infarction, carotid artery ligation-induced intimal hyperplasia and VSMC migration both *in vivo* and *in vitro*.

## Materials and Methods

### Chemicals and reagents

Pipoxolan was a gift from the Wide Pharmaceutical Co., Ltd. (Taichung, Taiwan) Pipoxolan was dissolved in dimethylsulfoxide (DMSO) (Sigma) and diluted in tissue culture medium before use. Zoletil^®^ was purchased from Virbac Laboratories (Carros, France). The chemicals were purchased from the following companies. PDGF-BB and MTT (3-[4,5-dimethylthiazol-2-yl]-2,5-diphenyl tetrazolium bromide) were from Sigma (St. Louis, MO, USA). Anti-Ras, anti-MEK, anti-phosphor-MEK1/2, anti-ERK, anti-phospho-ERK1/2, anti-MMP-2, and anti-MMP-9 were from Abcam (Cambridge, UK). Anti-β-actin was from Santa Cruz Biotechnology (Santa Cruz, CA, USA). DMEM, penicillin/streptomycin, FBS, and glutamine were from Thermo (Thermo Scientific Inc, Waltham, PA, USA).

### Animals

Male Sprague-Dawley (SD) rats, weighing 225–275 g and male ICR mice, weighing 25-30 g were purchased from BioLASCO Co. Ltd. (Taipei, Taiwan). All animals were fed regular chow and housed in standard cages at a constant temperature of 22 ± 1 °C with 12 hr inverted light–dark cycle for 1 week prior to the experiments. The experimental protocol was approved by the Committee on Animal Research, China Medical University (Permit Number: 99-12, 101-251). Surgery was performed under zoletil^®^ anesthesia. Five to six animals were used to obtain consistent data in each group.

### Transient focal cerebral ischemia–reperfusion model

Focal ischemia was induced by occlusion of both common carotid arteries (CCA) and the right middle cerebral artery (MCA) as previously described [24]. Eighteen rats were randomly assigned to 3 treatment groups, six rats for each group. Group 1 rats were treated with saline and groups 2 and 3 treated with PIPO (10 mg/kg and 30 mg/kg, respectively). Rats were fasted overnight with free access to water. PIPO (10, 30 mg/kg) was orally administered 1 hr prior to transient focal cerebral ischemia-reperfusion. Animals then were anesthetized with zoletil^®^ (25 mg/kg i.p.), and the skull was exposed, and a small burr hole was produced over the MCA. Beneath the right MCA, proximal to the major bifurcation of the right MCA, distal to the lenticulostriate arteries, and rostral to the rhinal fissure, a 10-0 nylon monofilament (Davis & Geck, Wayne, NJ, USA) was placed. The artery then was lifted, and the nylon filament rotated clockwise. Both CCA were then occluded using a microvascular clip (FE691; Aesculap, Tuttlingen, Germany). After 90 minutes of occlusion, reperfusion was established by removing the microvascular clips from the CCA, rotating the nylon monofilament counterclockwise and removing it from beneath the MCA. After 24 hr, all rats were alive and neurobehavioral evaluation and infarct assessment were performed.

### Direct blood pressure measurement and cerebral blood flow recording

Male Sprague-Dawley rats were anesthetized with zoletil^®^ (25mg/kg i.p.), and the right femoral artery was then exposed through a skin incision in the right inguinal region. The right femoral artery was cannulated and connected to a pressure transducer coupled to a PowerLab^®^ recording system, and an application program (Chart, V5.0; all from A D Instruments, Castle Hill, Australia) recording mean arterial pressure (MAP). Saline or PIPO (10, 30 mg/kg) was orally administered 60 min prior to blood pressure measurement. The cerebral blood flow (CBF) was recorded by a Laser Doppler cerebral blood flow monitoring apparatus (Laser Doppler Flowmetry, Oxford Optronix Ltd., Oxford, UK) which was connected to a laptop for data collection and analysis. CBF was measured by a data acquisition system (PowerLab^®^, Chart V5.0, A D Instruments, Castle Hill, Australia). The mean CBF at baseline was used as the denominator (100%) and compared to the mean CBF during the ischemia period and the reperfusion.

### Evaluation of neurological deficits and measurement of infarction

Twenty four hr following reperfusion, neurological function of rats was assessed as previously published [25,26]. The degree of neurological deficits were divided 0-5 (higher score is for more severe neurological deficits) as follows: 0 = no neurological deficits, 1 = failure to extend left forepaw fully, 2 = circling to the left, 3 = falling to left, 4 = no spontaneous walking with a depressed level of consciousness, and 5 = death. Neurological evaluation was done by an observer who was blind to the treatment conditions of the animals. After completion of the neurobehavioral evaluation, rats were deeply anesthetized by an intraperitoneal injection of zoletil^®^ (50 mg/kg) followed by intracardiac perfusion with 200 ml of freezing PBS and animals were then decapitated. The brain was removed and sliced in 2-mm sections using a rodent brain matrix slicer (RBM-4000C; ASI Instruments, Warren, MI, USA). The sections were stained with 2% 2,3,5-triphenyltetrazolium chloride (TTC) for 10 min at 37 °C and fixed in 10% formalin. The image of each section was digitized and the infarct area was determined morphometrically using Image-Pro Plus 6.0 (Media Cybernetics, MD, USA) [24].

### TUNEL assay

Nine rats were randomly assigned into 3 treatment groups, three rats for each group. Group 1 rats were treated with saline and groups 2 and 3 treated with PIPO (10 mg/kg and 30 mg/kg, respectively). Focal ischemia was induced by occlusion of both CCA and the right MCA as previously described [24]. Twenty four hr after focal ischemia, rats were deeply anesthetized by intraperitoneal injection of 50 mg/kg of zoletil^®^ followed by intracardiac perfusion with 200 ml of 0.9% saline, followed by 4% paraformaldehyde in 0.1 M PBS and animals were decapitated [27]. The percentage of positive TUNEL staining cells within areas of the cortex was estimated.

### Immunohistochemical staining of cleaved caspase-3

Rat brain slices were incubated with anti-caspase-3 antibodies (GTX73093, GeneTex Inc., USA) overnight and immunohistochemical labeled using a NovoLink Polymer Detection System Kit (Leica Microsystems Inc., Newcastle Upon Tyne, UK) as previously described [27]. The percentage of positive caspase-3 staining cells within the cortex was estimated based on the average number of cells in a defined area.

### Animal carotid ligation model

Male ICR mice, weighing 20 to 25 g, were used as a carotid-ligation model according to a previous report [28]. Fifteen mice were randomly assigned to 3 treatment groups, five mice for each group. All mice were fasted overnight with free access to water, then anesthetized with zoletil® (25 mg/kg i.p.). Carotid ligation was performed by a midline neck incision and the left common carotid artery exposed. The carotid artery was completely ligated just proximal to the carotid bifurcation, and the right carotid artery served as a non-injured control artery [29]. The incision was closed after ligation, and the animals were allowed to recover. On the day of carotid ligation, mice were randomized into 3 treatment groups and anesthetized as described. Group 1 mice were treated with saline and groups 2 and 3 treated with PIPO (10 mg/kg and 30 mg/kg, respectively). Subsequent PIPO and saline treatments were given by gastric gavage on the second day after carotid ligation and once daily for 28 days. All animals were euthanized on Day 29, and both right and left common carotid arteries were harvested, dehydrated in ethanol and xylene and embedded in paraffin for histomorphometric analysis.

### Hematoxylin-eosin staining and PCNA antibody staining of the carotid artery

Arterial sections (5 µm) of mice euthanized on Day 29 after ligation were stained by use of a hematoxylin-eosin solution as previously described [28]. Images were digitized and analyzed with Image-Pro software. The areas of the lumen, internal elastic lamina (IEL), and external elastic lamina (EEL) were determined by computerized planimetry. The intima area was calculated by subtracting the luminal from the IEL area, and media area was determined by subtracting the IEL area from the EEL area [29]. The ratio of intima to media area (I/M ratio) was calculated and analyzed. Arterial sections of animals from the different treatment groups were stained by using an immunofluorescence method to detect proliferating cell nuclear antigen (PCNA) [30].

### Vascular smooth cell line

The VSMC cell line A7r5 was purchased from Bioresource Collection and Research Center (Hsinchu, Taiwan). A7r5 cells were plated onto 6-well plates in DMEM, supplemented with 10% FBS, 100 units/ml penicillin, 100 µg/ml streptomycin and 2 mM L-glutamine and grown at 37 °C under a humidified 5% CO_2_ and 95% air at one atmosphere.

### In vitro wound healing assay

The effects of PIPO on VSMC migration were evaluated by a wound-healing assay. A7r5 cells were plated in 6-well plates (2x10^5^ cells per well) and damage was performed with a single scratch wound using a sterile micropipette tip as previously described [28]. Cells were then incubated with or without PDGF-BB (30 ng/ml) and PIPO (5, 10, 15 µM) in serum-reduced DMEM medium (containing 0.5% fetal bovine serum). The extent of wound closure was determined using a phase-contrast microscope at 24, 48, and 72 hr following wounding. Cell migration was expressed by the migration distance of drug-treated cells (mm) divided by the migration distance of untreated cells (mm) [28].

### Transwell migration assay

Effects of PIPO on VSMC migration were further investigated by a transwell migration chamber with a collagen-coated polycarbonate filter as previously described [28]. A7r5 cells (2x10^5^ cells) were seeded on a transwell apparatus (a 6.5-mm polyethylene terephthalate membrane with 8-µm pores; Millicell, Millipore Inc, Billerica, MA01821, USA) and treated with PIPO (0, 5, 10 and 15 µM) in the presence of PDGF-BB (30 ng/ml) for 48 hr. The cells were then trypsinized, re-suspended in 0.5% FBS medium. FBS/DMEM (10%) was added to the bottom chamber of each well as the chemo-attractant. Cells were allowed to migrate for 8 hr through the membrane to the underside of the apparatus. Cells were then fixed with methanol for 10 min and stained with Giemsa solution for 30min. The cells migrating to the lower outside of the insert membrane were counted manually using a microscope and the NIS-Elements software (Nikon Inc, Melville, NY, USA).

### Protein preparation and Western blot analysis

The role of matrix metalloproteinase-2/9 and the Ras/MEK/ERK signaling pathways were used to evaluate the effect of PIPO on VSMC migration. A7r5 cells (5 × 10^6^ cells) were seeded in a 10 cm culture dish and then treated with PDGF-BB (30 ng) and PIPO (5, 10, 15 µM) for 48 hr. The cells were harvested and washed with cold 1x PBS. Total protein concentration was measured using the BCA assay kit (Pierce Biotechnology Inc., Rockford, IL, USA). An equal volume of cell lysate was run on a 10% SDS-polyacrylamide gel electrophoresis (SDS-PAGE) and electrotransferred to a polyvinylidene fluoride membrane (PVDF, Thermo Scientific Inc, Waltham, PA, USA) by using iBot^TM^ Gel Transfer System (Invitrogen). The blot was soaked in blocking buffer (5% non-fat dry milk/0.05% Tween 20 in 20 mM TBS at pH 7.6), at room temperature for 1 hr, and then incubated with anti-Ras, anti-MEK, anti-phosphor-MEK1/2, anti-ERK, anti-phospho-ERK1/2, anti-MMP-2, anti-MMP-9 and β-actin antibodies in blocking buffer, respectively, at 4 °C overnight as previously described [28]. Membranes were washed with Tris-buffered saline/Tween 20 three times for 10 minutes, and then incubated with secondary horseradish peroxidase (HRP)-conjugated antibody. The blots were developed using a chemiluminescence (ECL) detection kit (Millipore, Billerica, MA, USA) as previously described [28]. β-Actin was used as an internal loading control.

### Statistical analysis

Data are expressed as mean ± SE. Differences among groups were analyzed using one-way analysis of variance (ANOVA) followed by Scheffe’s test for individual comparisons. A *p*-value of <0.05 was considered statistically significant.

## Results

### Effect of PIPO on mean arterial pressure and cerebral blood flow

To facilitate postoperative recovery additional surgical procedures were not used for physiological monitoring. However, we performed a separate experiment to investigate the effects of PIPO on major physiological parameters in ischemic rats. Saline or PIPO (10, 30 mg/kg) was orally administered 60 min prior to MCAO. Mean arterial pressure (MAP) and cerebral blood flow (CBF), blood glucose, rectal temperature, blood pH and blood gas (*p*O_2_ and *p*CO_2_) were measured before ischemia, during the occlusion and reperfusion. PIPO did not alter those physiological parameters when compared with control animals (data not shown). Moreover, MAP and CBF were not affected by PIPO (10, 30 mg/kg p.o.) administered 60 min prior to MCAO (Table 1).

**Table 1:** Cerebral blood flow and mean arterial pressure of rat in ischemia/reperfusion injury.

	**Cerebral blood flow (% of the baseline**)						**Mean arterial pressure (mmHg**)		
**I/R**	**Injured hemisphere**			**Non-injured hemisphere**					
**PIPO**	**0 mg/kg**	**10 mg/kg**	**30 mg/kg**	**0 mg/kg**	**10 mg/kg**	**30 mg/kg**	**0 mg/kg**	**10 mg/kg**	**30 mg/kg**
Before	100.0±0.0	100.0±0.0	100.0±0.0	100.0±0.0	100.0±0.0	100.0±0.0	140.4±2.8	135.7±1.7	142.2±0.8
During	4.8±0.7	6.6±1.0	6.8±2.4	99.5±1.3	98.5±0.8	101.2±2.6	137.5±3.1	136.0±2.7	142.1±2.6
After	115.5±5.0	108.9±6.4	112.7±4.2	100.5±1.2	103.7±3.2	102.6±2.8	136.3±2.6	137.1±4.6	139.6±3.0

1 I/R: ischemia/reperfusion injury

2 The cerebral blood flow before I/R is defined as the baseline (100%)

3 Pipoxolan (PIPO) was orally administered 60 min prior to I/R (n=3)

### Effects of PIPO on cerebral infarction area, TUNEL staining and cleaved caspase-3 expression in the brain of ischemic rats

**Figure 1:**
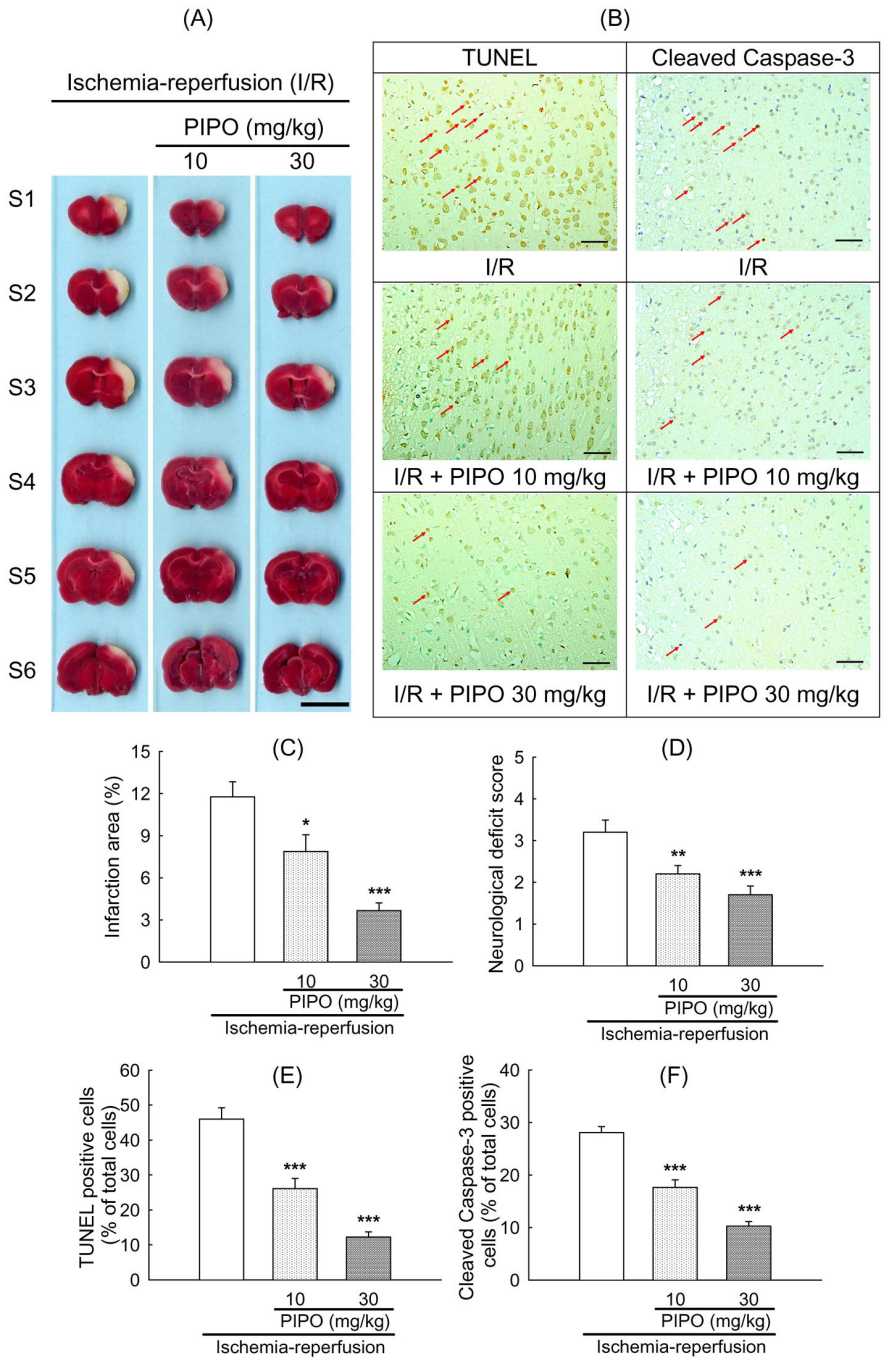
Effects of pipoxolan (PIPO) on cerebral infarction of rats. (A) Effects of PIPO (10, 30 mg/kg p.o.) groups on cerebral infarct area at 24 hr after reperfusion. The pale area represents infarct tissue and the red area normal tissue. Scale bar = 1 cm.(B) Representative micrographs of TUNEL and cleaved caspase-3 positive staining in ischemic rats treated with/without PIPO. Scale bar = 50 µm. Magnification 200x. (C) Infarction area by TTC staining, (D) neurological deficit score, (E) TUNEL-positive cells and (F) cleaved caspase-3- positive cells (n = 6 in each group). Each vertical bars represented mean ± S.E. *P < 0.05, **p<0.01 and ***p < 0.001 compared to I/R group.

Cerebral infarction in coronal sections was observed in the control group. PIPO treatment (10 and 30 mg/kg) significantly reduced cerebral infarction as compared with the control group (Figure 1A). Percent reduction of cerebral infarction by PIPO were 43.18% and 73.43% for 10 and 30 mg/kg, respectively (***p<0.001) (Figure 1C). The neurological deficits in rats were 3.20 ± 0.29, 2.20 ± 0.20, 1.7 ± 0.21, which was a reduction of 0, 31.25 and 46.88% by PIPO (0, 10 and 30 mg/kg p.o.), respectively Figure 1 (D) (**p<0.01, ***P< 0.001). Representative micrographs of TUNEL and cleaved caspase-3 positive staining in ischemic rats treated with/without PIPO were shown in Figure 1B. TUNEL and cleaved caspase-3 positive cells were significantly decreased by PIPO treatment compared with control cells. Percent reduction of TUNEL positive cells by PIPO were 31.25% (10 mg/kg) and 46.88% (30 mg/kg), respectively (Figure 1E). PIPO induced reduction of cleaved caspase 3 positive cells were 37.18% (10 mg/kg) and 63.44% (30 mg/kg), respectively (***p<0.001) (Figure 1F).

### Effects of PIPO on intimal hyperplasia and proliferation of cell nuclear antigen expression

**Figure 2:**
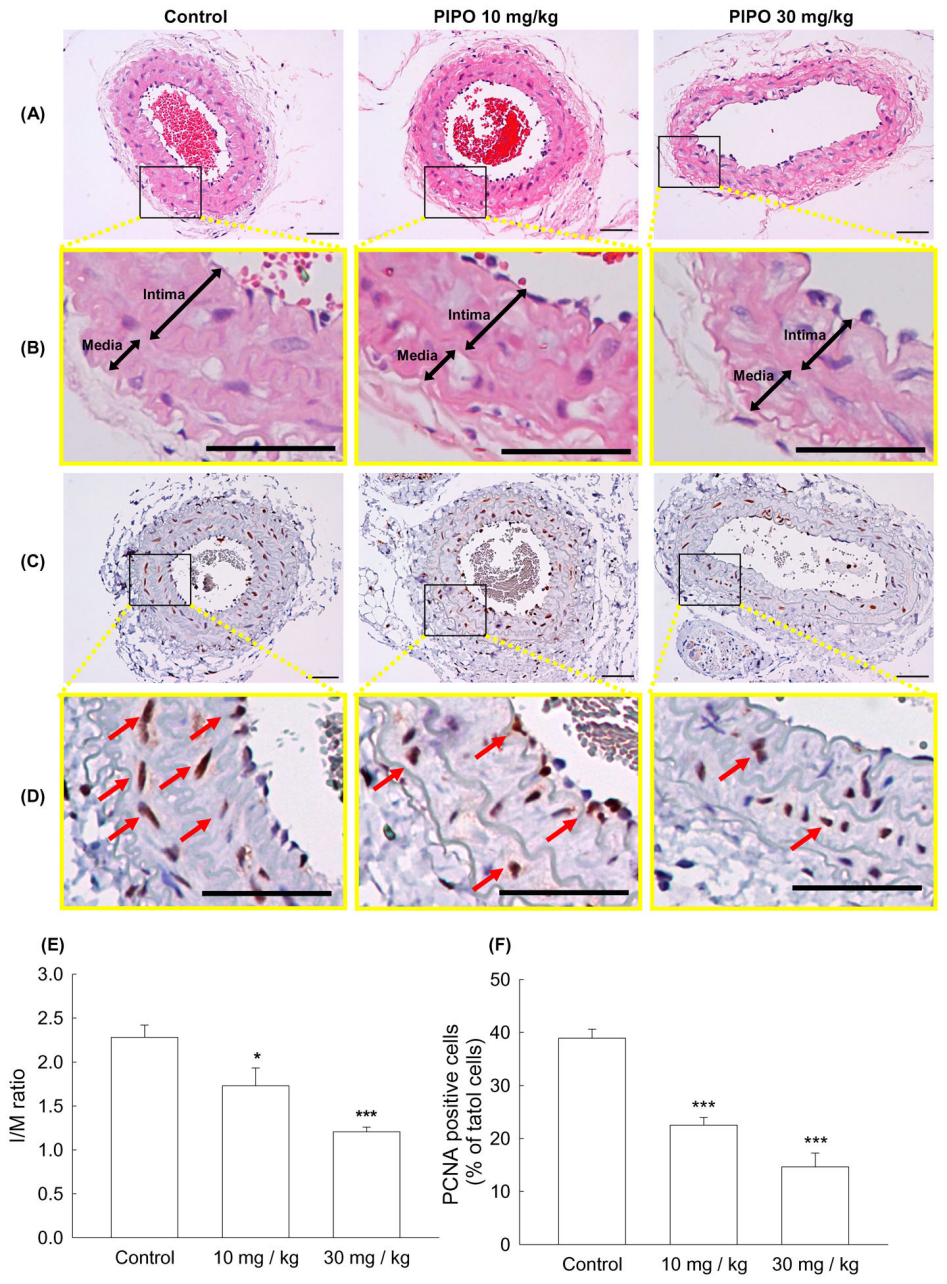
Effects of PIPO on carotid-ligation induced intimal hyperplasia in mice. Different doses of PIPO (10 and 30 mg/kg) was orally administered once a day for 28 days. (A) and (B) represent photomicrographs of hematoxylin-eosin staining, and (C) and (D) represents PCNA-immunoreactivity staining of arterial section (200 X). (E) I/M ratio. (F) Percentage of PCNA positive cells per total cells. The arrow was indicating the PCNA positive cell. Scale bar = 50 µm. *P< 0.05 and ***p< 0.001 compared with control group (n=5).

After carotid-ligation in mice receiving PIPO or saline for 4 weeks, intimal hyperplasia morphology was assessed by measuring the absolute lumen area, intima area (I), media area(M), and intima/media ratio (I/M). The morphology and I/M ratio were increased at 4 weeks following carotid-ligation. Mice treated with PIPO (10 mg/kg and 30 mg/kg p.o.) showed a significant reduction in carotid intimal hyperplasia compared with the control (saline) group. Percent reductions of I/M ratios were 24.21% (10 mg/kg, *p<0.05) and 47.20% (30 mg/kg, ***p<0.001) compared with the control group (Figure 2A, 2B and 2E). The number of proliferating cell nuclear antigen (PCNA)-positive cells was also inhibited by PIPO (10, 30 mg/kg) treatment. Percent inhibition was 42.11% (***p<0.001) and 62.40% (***p<0.001), respectively (Figure 2C, 2D and 2F).

### Effects of PIPO on VSMC migration

**Figure 3:**
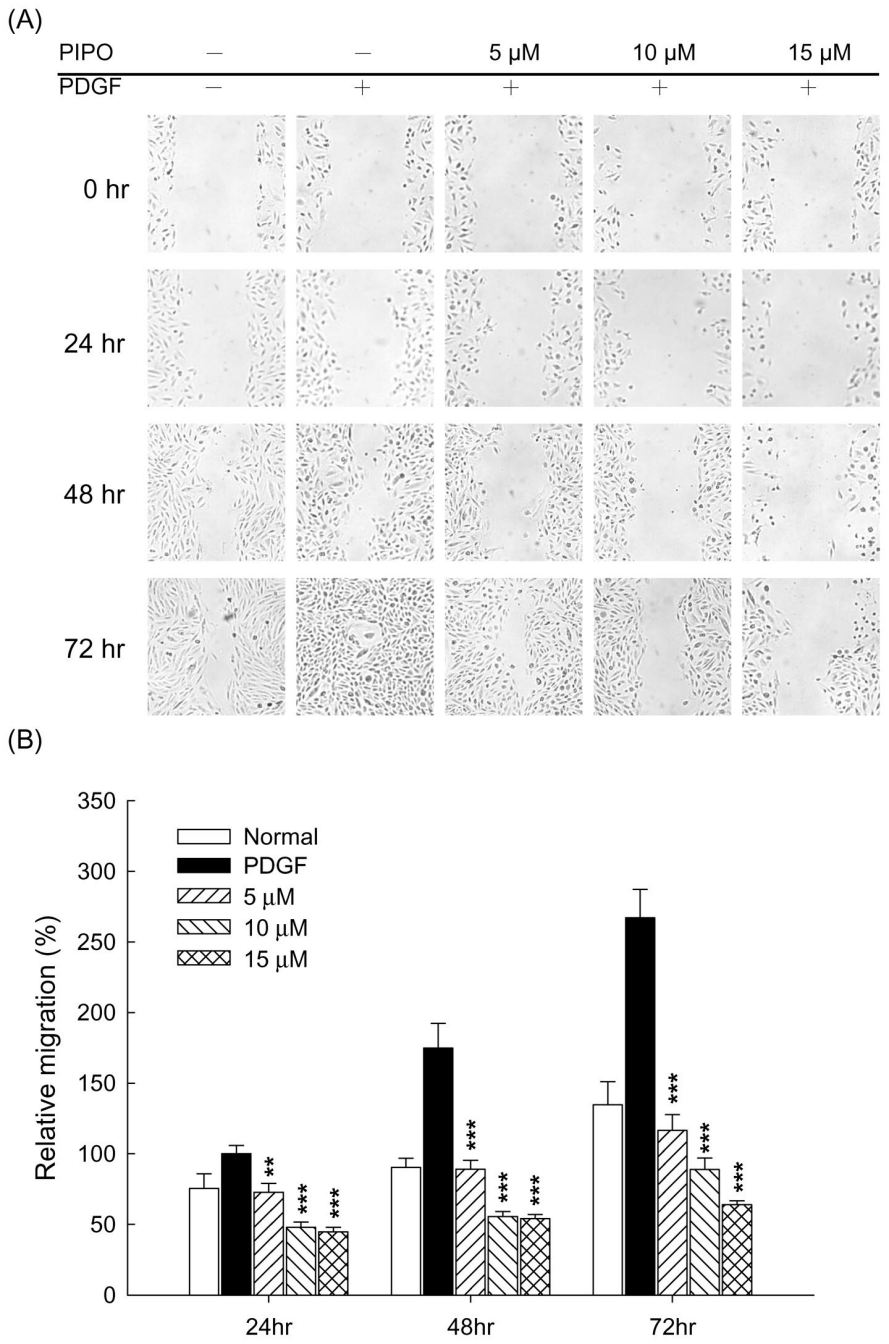
Effects of PIPO on PDGF-BB-induced VSMC migration by wound healing assay. (A) A typical trace of PIPO (5, 10, and 15 µM) inhibited in response to PDGF-BB-induced VSMC migration. (B) Statistical differences in 24, 48 and 72 hr at different PIPO concentrations, respectively. Normal group was treated with vehicle. **P<0.01 and ***p<0.001 compared with PDGF-BB control group (n=6)..

**Figure 4:**
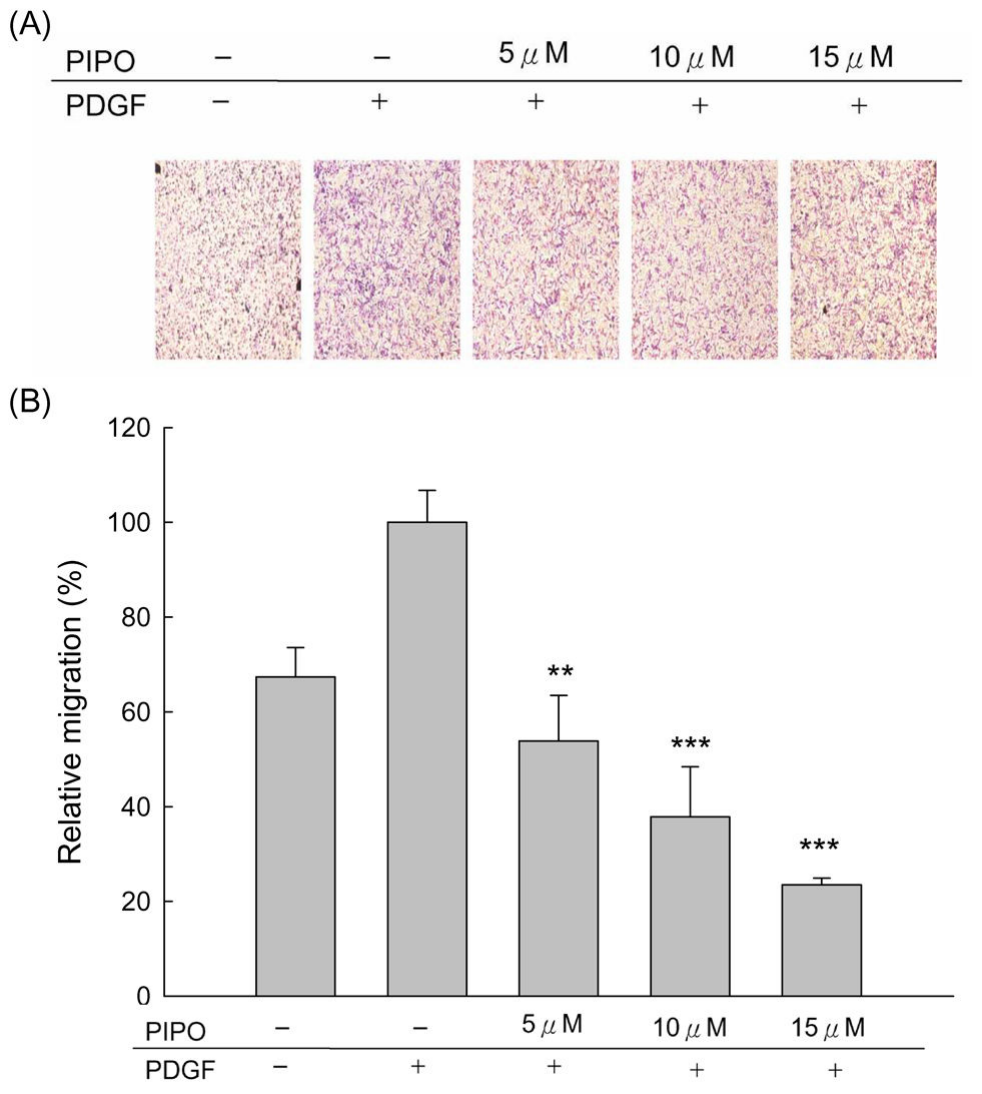
Effects of PIPO on PDGF-BB-induced VSMC migration by transwell assay. (A) Inhibition of PIPO (5, 10, and 15 µM) in response to PDGF-BB-induced VSMC migration. (B) Statistical differences of PIPO in response to PDGF-BB-induced VSMC migration at different PIPO concentrations. Normal group was treated with vehicle. **P <0.01 and ***p<0.001 compared with PDGF-BB control treated group (n=6)..

Incubation of A7r5 cells with PDGF-BB-stimulated migration, which was significantly attenuated by PIPO at 24, 48 and 72 hr. PIPO (5, 10 and 15 µM) markedly inhibited the PDGF-BB-stimulated cell migration (Figure 3A) in a dose-dependent manner (Figure 3B, ***p<0.001). The inhibitory effects on A7r5 cell migration were further confirmed by a transwell migration assay. Figure 4 shows that the ability of A7r5 cells to move across the membrane was significantly reduced by PIPO. Percent inhibition of cells treated with PIPO (5, 10 and 15 µM) was 46.2% (**p<0.01), 62.2% (***p<0.001) and 76.5% (***p<0.001) compared with the positive control (PDGF-BB) cells, respectively.

### Effects of PIPO on MMPs and Ras/MEK/ERK protein levels

**Figure 5:**
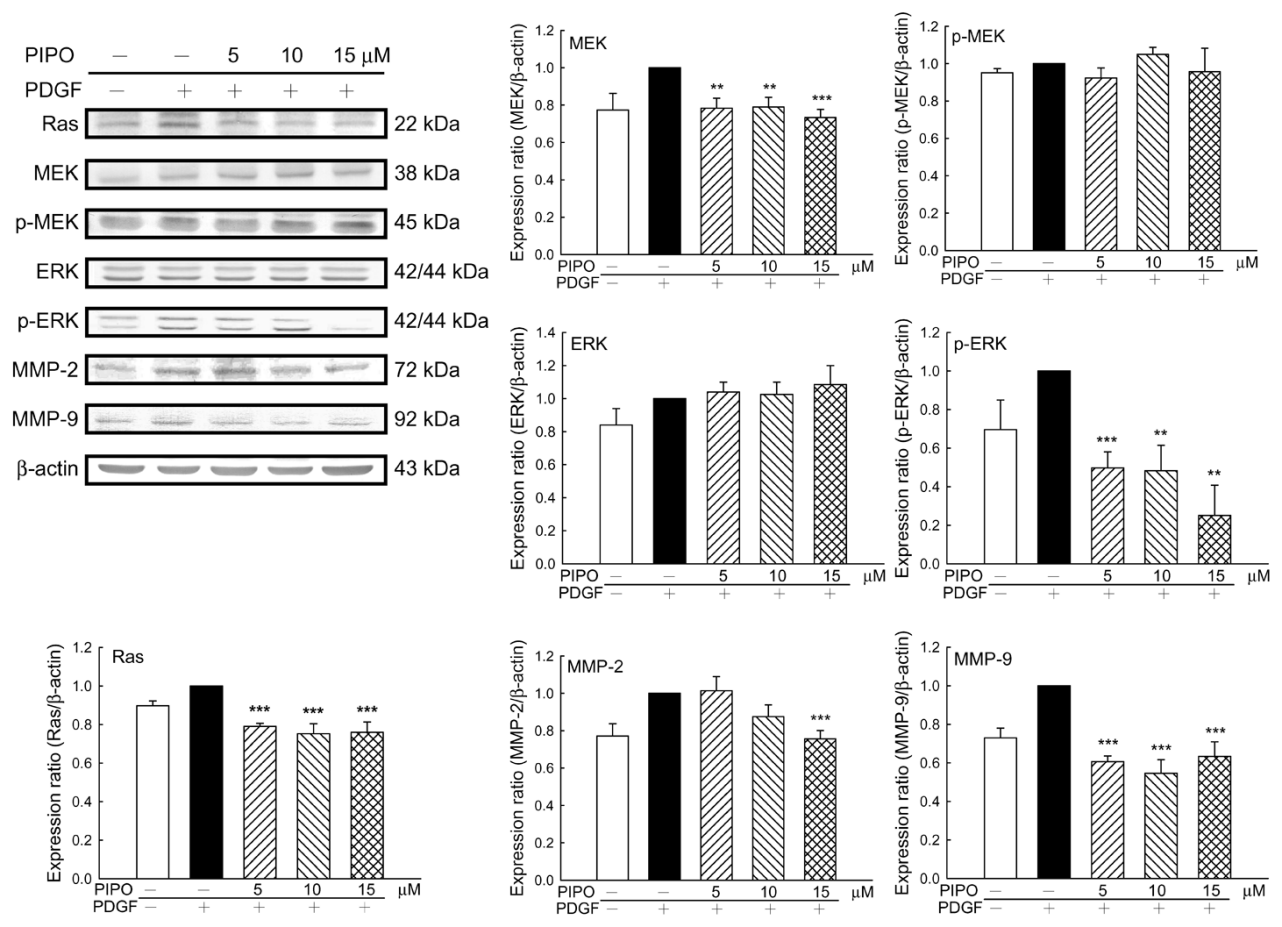
Effects of PIPO on protein expression level in PDGF-BB-induced VSMCs. The protein expression levels were determined by Western blot analysis. PIPO significantly decreased the expression levels of Ras, MEK, p-ERK, MMP-2, and MMP-9. **P<0.01, ***p<0.001 (n=6).

A series of experiments were performed to measure effects of PIPO on levels of the candidate signaling proteins matrix metalloproteinase-2/9, Ras, MEK, p-MEK, ERK and p-ERK in PDGF-BB-stimulated VSMCs. PIPO at a concentration of 5, 10, 15 µM significantly reduced protein levels of Ras (21%, 24.75% and 24%, respectively ***p<0.001); MEK (21.67%, 21% and 26.67%, respectively ***p<0.001); and p-ERK (50.33%, 51.67% and 75%, respectively ***p<0.001). MMP-2 protein levels were reduced by PIPO (10, 15 µM) 12.4% and 24.4%, respectively ***p<0.001. MMP-9 levels were significantly inhibited by PIPO (5, 10, 15 µM) at 39.4%, 45.4% and 36.6%, respectively (***p<0.001) (Figure 5).

## Discussion

Cerebral ischemia and infarction are most commonly associated with atherosclerotic disease and stroke in the carotid and vertebrobasilar circulation [28,31]. Prolonged vascular injury can lead to small vessel occlusion and cerebral infarction [1]. Blood vessels in response to injury or atherosclerosis usually display intimal thickening. The balance of migration and proliferation of vascular smooth cells (VSMC) over apoptosis impacts the final size of intimal thickening [7]. The purpose of the present study was to investigate effects of PIPO on cerebral ischemia and carotid artery ligation-induced intimal hyperplasia. PIPO treatment decreased the size of the ischemia/reperfusion induced cerebral infarction in a dose-dependent manner. Treatment with PIPO had a dose-dependent inhibitory effect on carotid artery ligation-induced intimal hyperplasia accompanied by a reduction of proliferating cell nuclear antigen (PCNA) expression. Moreover, PIPO (5~15 µM) did not affect the viability of the cultured VSMC cell line A7r5. Treatment with PIPO had a dose-dependent inhibitory effect on PDGF-BB- stimulated VSMC proliferation. These data indicate that PIPO can counter effects of cerebral ischemia.

Endothelial cells can synthesize and release catecholamines in response to ischemia [32]. Pipoxolan (PIPO) inhibited norepinephrine release and high K^+^-induced vasoconstriction. Vasodilation may reduce the pathogenesis of atherosclerosis and restenosis and prevent cerebral ischemia resulting from decreasing blood flow. Saline or PIPO (10, 30 mg/kg) was orally administered 60 min prior to MCAO. Mean arterial pressure (MAP) and cerebral blood flow (CBF), blood glucose, rectal temperature, blood pH and blood gas (*p*O_2_ and *p*CO_2_) were measured during occlusion and reperfusion before ischemia. The beneficial effects of PIPO were not related to modification of physiological parameters since those parameters did not differ between PIPO- and saline-treated rats.

Platelets, VSMCs and endothelial cells in the damaged vascular wall can induce PDGF-BB release. PDGF-BB is a potent growth factor that can initiate a variety of biological responses by activating intracellular signal transduction pathways such as MAPK/ERK, leading to vascular smooth muscle cell proliferation and migration [33]. VSMCs proliferation and migration are significant contributors to neointima formation after balloon injury. Modulation of VSMC growth has important therapeutic implications [34,35]. MAPK pathways play an influential role in promoting VSMC proliferation [36]. MAPK signaling pathways are activated by vascular injury and inhibition of the activated ERK pathway by drugs or gene therapy can reduce neointimal hyperplasia [28,37]. PIPO inhibited ERK phosphorylation. Protein levels of Ras and MEK which are associated with VSMC proliferation and migration were reduced by PIPO treatment. Ras/MEK/ERK signaling pathways are involved in cardiovascular disease [38]. PIPO significantly reduced protein levels of MMP-2 and MMP-9. MMPs regulate the activity of VSMC *in vitro* and *in vivo* [7]. MMPs, with matrix degrading activity induced VSMC proliferation and migration [39]. MMP-2 and MMP-9 are up-regulated in human atherosclerotic plaques [40]. Results from this study revealed that the inhibition of PIPO (5, 10, 15 µM) on protein levels of Ras and MMP-9 in PDGF-BB-induced VSMCs reached a maximal effect even in a lower dose (5 µM). The functional measurement *in vivo* and *in vitro*, such as infarction area, TUNEL positive cells, cleaved caspase 3-positive cells, carotid-ligation induced intimal hyperplasia, wound healing assay or transwell assay is correlated to complex interactions between several signaling pathways. PIPO treatment did reduce the cerebral infarction area; neurological deficits and carotid-ligation induced intimal hyperplasia *in vivo* and inhibited VSMC migration *in vitro*, in a dose-dependent manner. Biological effects of PDGF-BB-induced experimental intimal hyperplasia exhibit similar characteristics as VSMC proliferation and migration after arterial injury [41]. Increasing gelatinase expression promotes the migration of VSMCs from the media area into the intima after vascular injury, leading to intimal hyperplasia and the restriction of normal blood flow [16]. Previous studies indicate that the gelatinases, MMP-2, MMP-9 are able to degrade the basement membrane, and promote intimal hyperplasia in animal models [42-44].

PIPO significantly reduced the severity of intimal hyperplasia in a carotid-ligation model and reduced the number of proliferating cell nuclear antigen (PCNA) -positive nucleoli. The PCNA can be used as an index of cell proliferation, and it has been identified by immunofluorescence as a polymerase-associated protein [30]. During the formation of atherosclerosis and intimal hyperplasia, various cytokines and growth factors stimulate VSMC to express MMPs. PIPO reduced the severity of intimal hyperplasia induced by carotid-ligation which was accompanied with the reduction of PCNA expression and PDGF-BB-stimulated VSMC migration. These effects were due to decreasing the activities of MMP-2 and MMP-9 and modulating the Ras/MEK/ERK signaling pathways.

In conclusion, PIPO is protective in models of ischemia/reperfusion-induced cerebral infarction, carotid artery ligation-induced intimal hyperplasia and VSMC migration both *in vivo* and *in vitro*. This multi-faceted effect of PIPO could be potentially efficacious in preventing cerebrovascular disease in high risk individuals, and it could be useful in treating patients with vascular disease.
